# Long-term Outcomes of Stereotactic Body Radiotherapy for Low-Risk and Intermediate-Risk Prostate Cancer

**DOI:** 10.1001/jamanetworkopen.2018.8006

**Published:** 2019-02-08

**Authors:** Amar U. Kishan, Audrey Dang, Alan J. Katz, Constantine A. Mantz, Sean P. Collins, Nima Aghdam, Fang-I Chu, Irving D. Kaplan, Limor Appelbaum, Donald B. Fuller, Robert M. Meier, D. Andrew Loblaw, Patrick Cheung, Huong T. Pham, Narek Shaverdian, Naomi Jiang, Ye Yuan, Hilary Bagshaw, Nicolas Prionas, Mark K. Buyyounouski, Daniel E. Spratt, Patrick W. Linson, Robert L. Hong, Nicholas G. Nickols, Michael L. Steinberg, Patrick A. Kupelian, Christopher R. King

**Affiliations:** 1 Department of Urology, University of California, Los Angeles; 2 Department of Radiation Oncology, University of California, Los Angeles; 3 Flushing Radiation Oncology Services, Flushing, New York; 4 21st Century Oncology, Fort Myers, Florida; 5 Department of Radiation Oncology, Georgetown University, Washington, DC; 6 Beth Israel Deaconess Medical Center, Harvard Medical School, Boston, Massachusetts; 7 Division of Genesis Healthcare Partners Inc, CyberKnife Centers of San Diego Inc, San Diego, California; 8 Swedish Radiosurgery Center, Seattle, Washington; 9 Department of Radiation Oncology, Odette Cancer Centre, Sunnybrook Health Sciences Centre, Toronto, Ontario, Canada; 10 Section of Radiation Oncology, Virginia Mason Medical Center, Seattle, Washington; 11 Now with Department of Radiation Oncology, Memorial Sloan Kettering Cancer Center, New York, New York; 12 Department of Radiation Oncology, Stanford University School of Medicine, Stanford, California; 13 Department of Radiation Oncology, University of Michigan, Ann Arbor; 14 Scripps Health, La Jolla, California; 15 Virginia Hospital Center, Arlington

## Abstract

**Importance:**

Stereotactic body radiotherapy harnesses improvements in technology to allow the completion of a course of external beam radiotherapy treatment for prostate cancer in the span of 4 to 5 treatment sessions. Although mounting short-term data support this approach, long-term outcomes have been sparsely reported.

**Objective:**

To assess long-term outcomes after stereotactic body radiotherapy for low-risk and intermediate-risk prostate cancer.

**Design, Setting, and Participants:**

This cohort study analyzed individual patient data from 2142 men enrolled in 10 single-institution phase 2 trials and 2 multi-institutional phase 2 trials of stereotactic body radiotherapy for low-risk and intermediate-risk prostate cancer between January 1, 2000, and December 31, 2012. Statistical analysis was performed based on follow-up from January 1, 2013, to May 1, 2018.

**Main Outcomes and Measures:**

The cumulative incidence of biochemical recurrence was estimated using a competing risk framework. Physician-scored genitourinary and gastrointestinal toxic event outcomes were defined per each individual study, generally by Radiation Therapy Oncology Group or Common Terminology Criteria for Adverse Events scoring systems. After central review, cumulative incidences of late grade 3 or higher toxic events were estimated using a Kaplan-Meier method.

**Results:**

A total of 2142 men (mean [SD] age, 67.9 [9.5] years) were eligible for analysis, of whom 1185 (55.3%) had low-risk disease, 692 (32.3%) had favorable intermediate-risk disease, and 265 (12.4%) had unfavorable intermediate-risk disease. The median follow-up period was 6.9 years (interquartile range, 4.9-8.1 years). Seven-year cumulative rates of biochemical recurrence were 4.5% (95% CI, 3.2%-5.8%) for low-risk disease, 8.6% (95% CI, 6.2%-11.0%) for favorable intermediate-risk disease, 14.9% (95% CI, 9.5%-20.2%) for unfavorable intermediate-risk disease, and 10.2% (95% CI, 8.0%-12.5%) for all intermediate-risk disease. The crude incidence of acute grade 3 or higher genitourinary toxic events was 0.60% (n = 13) and of gastrointestinal toxic events was 0.09% (n = 2), and the 7-year cumulative incidence of late grade 3 or higher genitourinary toxic events was 2.4% (95% CI, 1.8%-3.2%) and of late grade 3 or higher gastrointestinal toxic events was 0.4% (95% CI, 0.2%-0.8%).

**Conclusions and Relevance:**

In this study, stereotactic body radiotherapy for low-risk and intermediate-risk disease was associated with low rates of severe toxic events and high rates of biochemical control. These data suggest that stereotactic body radiotherapy is an appropriate definitive treatment modality for low-risk and intermediate-risk prostate cancer.

## Key Points

**Question:**

Is stereotactic body radiotherapy safe and effective in the long term for low-risk and intermediate-risk prostate cancer?

**Findings:**

In this pooled, individual patient data analysis of cohort studies with a total of 2142 patients with low-risk and intermediate-risk prostate cancer treated with stereotactic body radiotherapy across 10 institutional studies and 2 multi-institutional trials, the 7-year incidence of biochemical recurrence was 4.5% for those with low-risk disease and 10.2% for those with intermediate-risk disease. The 7-year cumulative incidence of severe genitourinary toxic events was 2.4% and of severe gastrointestinal toxic events was 0.4%.

**Meaning:**

These findings suggest that stereotactic body radiotherapy is associated with a long-term clinical outcomes profile—both in terms of toxic events and disease control—that is comparable with other, more widely used treatments for low-risk and intermediate-risk prostate cancer.

## Teaser

This cohort study of pooled individual patient data assesses long-term outcomes after stereotactic body radiotherapy for low-risk and intermediate-risk prostate cancer.

## Introduction

Prostate cancer (PCa) is the leading cause of cancer treatment–related years lived with disability worldwide, reflecting the confluence of its high incidence, high cure rate, and treatment-associated morbidity.^1^ Most patients with PCa in the developed world receive a diagnosis of clinically localized disease, and the majority have low-risk or intermediate-risk disease as defined by the National Comprehensive Cancer Network (NCCN).^2^ Multiple management options are available, including definitive external beam radiotherapy (EBRT), radical prostatectomy, brachytherapy, and (for patients with low-risk and favorable intermediate-risk disease) active surveillance.

Traditionally, definitive EBRT has been delivered in small fractions of 1.8 to 2.0 Gy spread across 8 to 9 weeks. Considerable preclinical and clinical data suggest that PCa specifically may exhibit an enhanced sensitivity to higher doses per fraction by virtue of a low α to β ratio (a proxy of radiosensitivity).^3^ A significant implication is that hypofractionation—treating with higher doses per fraction—may allow, at the least, isoeffective oncologic results in a shorter time frame. Moderate hypofractionation (using fractions of 2.5-3.0 Gy) has been studied extensively, with 3 noninferiority randomized clinical trials demonstrating the efficacy and safety of this approach.^4,5,6^

Stereotactic body radiotherapy (SBRT), or extreme hypofractionation, is a specific form of EBRT in which advanced radiotherapy techniques are used to deliver very large doses of radiation per fraction. Since its inception in 2000, several single-institution trials and 2 multi-institutional trials of SBRT for PCa have been reported, generally with median follow-up periods of 3 to 5 years.^7^ A large, multi-institutional consortium report of 1100 patients with a median follow-up of 3 years presented 5-year biochemical relapse–free survival rates of 95% for patients with low-risk disease and 84% for patients with intermediate-risk disease.^8^ Based on the overall favorable outcomes of these studies, the NCCN guidelines since 2014 have stated that SBRT “can be considered as an alternative to conventionally fractionated regimens at clinics with appropriate technology, physics, and clinical expertise.”^2^^(pMS-17)^ Stereotactic body radiotherapy regimens have been associated with reduced cost^9,10^ and less regret about undergoing treatment^11^ compared with other radiotherapy regimens.

However, there have been concerns raised in both the academic literature^12,13,14^ and the lay press^15^ about long-term outcomes and, specifically, long-term toxic effects of SBRT. In fact, the NCCN guidelines continue to state that “longer follow-up and prospective multi-institutional data are required to evaluate longer-term results.”^2^^(pMS-17)^ The aims of the present study were to evaluate the long-term outcomes associated with SBRT in a large cohort of 2142 men with low-risk PCa and intermediate-risk PCa treated prospectively with SBRT across multiple institutions between 2000 and 2012.

## Methods

### Study Design and Participants

Eligible patients were identified by querying 10 single-institution phase 2 trials and 2 multicenter prospective phase 2 trials (NCT00643994 and NCT00643617; Table 1).^16,17,18,19,20,21,22,23,24,25,26^ All patients received treatment between January 1, 2000, and December 31, 2012, for low-risk PCa or intermediate-risk PCa per the NCCN risk stratification scheme.^2^ Intermediate-risk disease was further stratified into favorable and unfavorable intermediate-risk groups, with the latter category reserved for patients with primary Gleason pattern 4 disease, multiple intermediate-risk factors, or 50% or more positive cores.^27^ Deidentified data were shared in concordance with the Health Insurance Portability and Accountability Act, with each institution’s institutional review board (UCLA [University of California, Los Angeles], Winthrop University Hospital, 21st Century Oncology, Georgetown University, Beth Israel Deaconess Medical Center, Genesis Healthcare Partners Inc, Swedish Medical Center, Sunnybrook Health Sciences Centre, Virginia Mason Medical Center, Stanford University, Scripps Health, and Virginia Hospital Center) approving contribution of data to the coordinating data center (UCLA) and waiving the need for patient informed consent. Abstraction and reporting of data from the compiled studies is in accordance with the Strengthening the Reporting of Observational Studies in Epidemiology (STROBE) reporting guideline for cohort studies.

**Table 1.** Individual Prospective Study Characteristics

Source	Years Treated	No. of Patients	Follow-up, Median (Range), y	Dose/Fraction (% of Patients Who Received Dose/Fraction)	Prescription Specification, %	Risk Group, %	Original Toxic Event Scoring
Masen et al,^16^ 2007	2000-2004	40	5.9 (0.7-15.0)	6.7 Gy ×5	90 Of prescribed dose to cover 100 of GTV	100 Low	RTOG and CTC v 2.0
King et al,^17^ 2012	2003-2009	67	9.5 (3.3-13.3)	7.25 Gy ×5	100 Of prescribed dose to cover 95 of PTV	73 Low, 15 Fav Int, and 2 Unfav Int	RTOG
Katz and Kang,^18^ 2014	2006-2010	477	7.9 (0.5-9.9)	7 Gy ×5 (32) and 7.25 Gy ×5 (68)	100 Of prescribed dose to cover 95 of PTV	68 Low, 22 Fav Int, and 9.8 Unfav Int	RTOG
Mantz,^19^ 2014	2007-2012	415	7.7 (5.0-10.4)	8 Gy ×5	100 Of prescribed dose to cover 98 of PTV	68.2 Low, 27 Fav Int, and 5 Unfav Int	CTCAE v 3.0
Meier et al,^20^ 2018	2008-2011	141	5.0 (0.1-8.2)	7.25 Gy ×5	100 Of prescribed dose to cover 95 of PTV	35 Low, 33 Fav Int, and 31 Unfav Int	CTCAE v 4.0
Fuller et al,^21^ 2018	2007-2012	206	5.0 (0.1-9.6)	9.5 Gy ×4	100 Of prescribed dose to cover 95 of PTV	43 Low, 35 Fav Int, and 21 Unfav Int	CTCAE v 4.0
Alayed et al,^22^ 2018	2006-2008	84	9.6 (1.0-10.8)	7 Gy ×5	95 Of prescribed dose to cover 99 of PTV	100 Low	CTCAE v 3.0
Alayed et al,^22^ 2018	2010	30	6.8 (5.7-7.2)	8 Gy ×5	95 Of prescribed dose to cover 99 of PTV	60 Low, 30 Fav Int, and 10 Unfav Int	CTCAE v 3.0
McBride et al,^23^ 2012	2006-2011	135	6.3 (0.1-10.3)	7.25 Gy ×5	100 Of prescribed dose to cover 95 of PTV	35 Low, 31 Fav Int, and 34 Unfav Int	CTCAE v 4.0
UCLA^24^	2010-2012	95	6.0 (0.3-8.1)	8 Gy ×5	100 Of prescribed dose to cover 95 of PTV	91 Low, 5 Fav Int, and 4 Unfav Int	CTCAE v 4.0
Fuller et al,^25^ 2014	2006-2012	51	6.0 (1.7-10.1)	9.5 Gy ×4	100 Of prescribed dose to cover 95 of PTV	1 Low, 71 Fav Int, and 28 Unfav Int	CTCAE v 3.0
Kataria et al,^26^ 2017	2007-2012	402	4.3 (1.8-9.1)	7 Gy ×5 (33) and 7.25 Gy ×5 (67)	100 Of prescribed dose to cover 95 of PTV	36 Low, 48 Fav Int, and 16 Unfav Int	CTCAE v 4.0 only for grade ≥3 toxic events^a^
Total	2000-2012	2142	6.9 (0.1-15.0)	NA	NA	65 Low, 25 Fav Int, and 9.9 Unfav Int	NA

Abbreviations: CTC v 2.0, Common Toxicity Criteria, version 2.0; CTCAE v 3.0 or v 4.0, Common Terminology Criteria for Adverse Events, version 3.0 or 4.0; Fav Int, favorable intermediate-risk disease; GTV, gross tumor volume; NA, not applicable; PTV, planning target volume; RTOG, Radiation Therapy Oncology Group; UCLA, University of California, Los Angeles; Unfav Int, unfavorable intermediate-risk disease.

^a^ This prospective study did not track physician-scored toxic events if they were less than grade 3 in severity by CTCAE v 4.0.

Doses of SBRT ranged from 33.5 to 40.0 Gy in 4 to 5 fractions (with 1885 of 2142 patients [88.0%] receiving 5 fractions). Treatments were delivered on consecutive days, every other day, or once a week per individual protocol specifications. Stereotactic body radiotherapy was delivered with either a robotic arm–mounted linear accelerator (CyberKnife; Accuray Inc) or a gantry-mounted linear accelerator. Specific treatment planning and delivery information is presented in eTable 1 in the Supplement. Patients were followed up with clinical evaluations performed and prostate-specific antigen level measured every 3 to 6 months for the first 2 years followed by every 6 to 12 months for the next 3 years.

### End Points

The cumulative incidence of biochemical recurrence (BCR) was the primary disease control end point, with BCR determined using the Phoenix definition of a prostate-specific antigen level of 2 ng/mL or more higher than the lowest post-SBRT value.^28^ Secondary disease control end points included the cumulative incidence of distant metastases (DMs), BCR-free survival, and overall survival. Physician-scored toxic event outcomes were scored prospectively as per the original trial criteria, focusing on genitourinary (GU) and gastrointestinal (GI) toxic events. Scoring criteria for toxic events were based on Common Toxicity Criteria, version 2.0^29^; Common Terminology Criteria for Adverse Events (CTCAE), version 3.0^30^ or version 4.0^31^; and/or the Radiation Therapy Oncology Group (RTOG) criteria^32^ (eTable 2 in the Supplement). An acute toxic event was defined as an adverse event occurring within the first 90 days after completion of SBRT. All instances of acute and late grade 3 or higher toxic events were centrally reviewed by 2 of us (A.U.K. and C.R.K.). No toxic events were downgraded, even if scoring by the current standard (CTCAE, version 4.0) would have allowed downgrading.

### Statistical Analysis

Statistical analysis was performed based on follow-up from January 1, 2013, through May 1, 2018. A competing risk framework was used to estimate the cumulative incidences of BCR and DMs, with death by any cause as a competing risk.^33^ Kaplan-Meier analysis was performed to estimate BCR-free survival and overall survival, with time to event set using the final day of SBRT as the starting point. This framework was also used to estimate the cumulative incidence of grade 3 or higher GU or GI toxic events. Univariate and multivariable Fine-Gray competing risk and Cox proportional hazards regression models were developed to assess the association between time to BCR and equivalent dose in 2-Gy fractions (EQD_2_) (calculated assuming an α to β ratio of 1.5), age, clinical T stage, ln (initial prostate-specific antigen level), use of androgen deprivation therapy, and Gleason grade group. The Cox proportional hazards regression models were used as the hazard ratios derived from such an analysis are easily interpretable, whereas competing risk regressions were used as it was thought that a competing risk framework more accurately modeled the relevant incidence of BCR. The proportional hazard test based on scaled Schoenfeld residuals was used to examine proportional hazard assumption. Univariate and multivariable logistic regression approaches were used to evaluate the association between the toxic event outcomes and EQD_2_, daily vs every-other-day fractionation, and treatment platform. To account for residual ecological bias, presuming patients enrolled in the same institutional study were likely to be more similar to each other than to patients enrolled in other institutional studies, the trial in which patients were enrolled was also included as a stratification factor in both the Cox proportional hazards regression and Fine-Gray competing risk models and as a random effect in the logistic regression models. Analyses were completed using R, version 3.3.2.^34^ All *P* values were from 2-tailed tests, and results were deemed statistically significant at *P* < .05.

## Results

Patient and treatment characteristics are presented in Table 2. A total of 2142 patients (mean [SD] age, 67.9 [9.5] years) were eligible for analysis, of whom 1185 (55.3%) had low-risk disease, 692 (32.3%) had favorable intermediate-risk disease, and 265 (12.4%) had unfavorable intermediate-risk disease. The percentage of positive cores was not available for 248 of 957 patients (25.9%) with intermediate-risk disease who did not have other criteria for unfavorable intermediate-risk disease; these patients were classified conservatively as having favorable intermediate-risk disease. The median follow-up period overall was 6.9 years (interquartile range [IQR], 4.9-8.1 years), and follow-up periods by risk group were as follows: low-risk, 7.1 years (IQR, 5.4-8.8 years); favorable intermediate-risk, 6.2 years (IQR, 4.1-7.9 years); and unfavorable intermediate-risk, 5.9 years (IQR, 3.3-7.1 years). Assuming an α to β ratio of 1.5 Gy, 797 patients (37.2%) received an EQD_2_ of 91 Gy or more. Overall, 115 men (5.4%) received concurrent androgen deprivation therapy, with rates ranging from 3.6% (43 of 1185) in patients with low-risk disease to 9.4% (25 of 265) in patients with unfavorable intermediate-risk disease. The median duration of androgen deprivation therapy was 3.6 months (range, 1-36 months).

**Table 2.** Patient and Treatment Characteristics

Characteristic	Value (N = 2142)
Age	
Mean (SD), y	67.9 (9.5)
Median (range), y	68 (41-92)
Risk grouping, No. (%)	
Low risk	1185 (55.3)
Favorable intermediate risk	692 (32.3)
Unfavorable intermediate risk^a^	265 (12.4)
Gleason grade, No. (%)	
I	1355 (63.3)
II	614 (28.7)
III	173 (8.1)
Clinical T stage, No. (%)	
T1c	1595 (74.5)
T2a	430 (20.1)
T2b	104 (4.9)
T2c	13 (0.6)
Initial prostate-specific antigen level	
Mean (SD), ng/mL	6.4 (3.1)
Median (range), ng/mL	5.7 (0.09-19.9)
Equivalent dose in 2-Gy fractions, No. (%)	
≥91 Gy	797 (37.2)
<91 Gy	1346 (62.8)
Treatment platform, No. (%)	
Robotic arm–mounted linear accelerator^b^	1479 (69.0)
Gantry-mounted linear accelerator	664 (31.0)
Fractionation, No. (%)	
Daily	1013 (47.3)
Every other day	1015 (47.4)
Weekly	114 (5.3)
Androgen deprivation therapy use, No./total No. (%)	
Total	115/2142 (5.4)
Low	43/1185 (3.6)
Favorable	47/692 (6.8)
Unfavorable	25/265 (9.4)
Duration of androgen deprivation therapy, mean (SD), mo	3.6 (4.2)

^a^ Percentage of positive cores was not available for 248 of 957 intermediate-risk patients (25.9%) who did not have other factors that could classify them as having unfavorable intermediate-risk disease; in instances of ambiguity, patients were classified conservatively as having favorable intermediate-risk disease.

^b^ CyberKnife (Accuray Inc).

**Figure 1.**
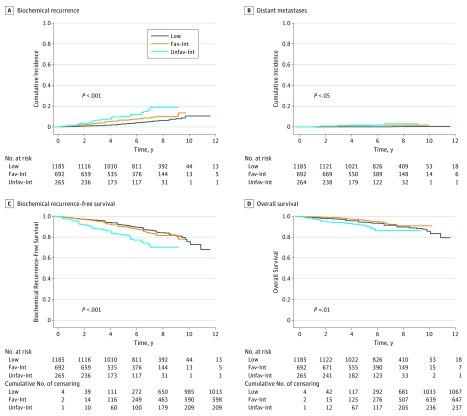
Study Outcomes A, Cumulative incidence of biochemical recurrence (*P* < .001). B, Cumulative incidence of distant metastases (*P* = .03). C, Kaplan-Meier curve of biochemical recurrence–free survival (*P* < .001). D, Kaplan-Meier curve of overall survival (*P* = .01). Fav-Int indicates favorable intermediate-risk disease; Low, low-risk disease; and Unfav-Int, unfavorable intermediate-risk disease.

Cumulative incidence plots of BCR and DM and Kaplan-Meier curves of BCR-free survival and overall survival are shown in Figure 1 and estimates are presented in eTable 3 in the Supplement. A total of 67 patients with low-risk disease developed BCRs, with a 7-year cumulative BCR incidence of 4.5% (95% CI, 3.2%-5.8%). Five patients developed metastases, corresponding to a 7-year DM rate of 0.1% (95% CI, 0.0%-0.3%). Among patients with favorable intermediate-risk disease, 51 developed BCR and 10 developed DMs, with a 7-year BCR rate of 8.6% (95% CI, 6.2%-11.0%) and a 7-year DM rate of 1.7% (95% CI, 0.6%-2.8%). Twenty-eight patients with unfavorable intermediate-risk disease developed BCRs and 4 developed DMs, with a 7-year BCR rate of 14.9% (95% CI, 9.5%-20.2%) and a 7-year DM rate of 3.0% (95% CI, 0.1%-5.8%). The 7-year BCR rate for all intermediate-risk disease was 10.2% (95% CI, 8.0%-12.5%), and the 7-year DM rate for all intermediate-risk disease was 2.0% (95% CI, 1.0%-3.0%). Overall, no patients died of prostate cancer. The 7-year overall survival rate for patients with low-risk disease was 91.4% (95% CI, 89.4%-93.0%), for patients with favorable intermediate-risk disease was 93.7% (95% CI, 91.0%-95.6%), for patients with unfavorable intermediate-risk disease was 86.5% (95% CI, 80.6%-90.7%), and for all patients with intermediate-risk disease was 91.7% (95% CI, 89.2%-93.6%). Neither EQD_2_ nor use of androgen deprivation therapy were significantly associated with time to BCR for any risk group with either competing risk regression or Cox proportional hazards regression modeling (eTables 4 and 5 in the Supplement).

Crude rates and cumulative incidence estimates of acute grade 2 and grade 3 or higher GU and GI toxic events and late grade 2 and grade 3 or higher GU and GI toxic events are shown in Table 3; narrative descriptions of grade 3 or higher toxic events are provided in eTable 6 in the Supplement. One participating institution, Georgetown, did not provide data for toxic events of grade 2 or lower, as toxic events were not a tracked end point in that institutional study. Thirteen patients experienced acute grade 3 or higher GU toxic events (crude incidence, 0.60%), and 2 others experienced acute grade 3 or higher GI toxic events (crude incidence, 0.09%). The most common acute grade 3 or higher GU toxic event was urinary frequency, which accounted for 8 events (61.5%). Among these, 2 patients (25.0%) had a prior cystoscopy and 3 (37.5%) developed late grade 3 or higher urinary frequency. The CTCAE, version 4.0 scale does not provide scores higher than 2 for urinary frequency; thus, these events were originally scored via RTOG and CTCAE, version 3.0, representing urinary frequency of once or more per hour or necessitating catheter placement.

**Table 3.** Crude Incidence of Acute Composite Radiation Therapy Oncology Group and Common Terminology Criteria for Adverse Events Grade 2 and Grade ≥3 Toxic Events, and Cumulative Incidence of Late Composite Radiation Therapy Oncology Group and Common Terminology Criteria for Adverse Events Grade 2 and Grade ≥3 Toxic Events^a^

Toxic Event	Crude Incidence, No. (%)^b^	Cumulative Incidence Estimate (95% CI)		
		5 y	7 y	10 y
Grade 2				
Acute GU	153 (9.0)	NA	NA	NA
Acute GI	56 (3.3)	NA	NA	NA
Late GU	163 (9.6)	11.2 (9.7-12.8)	12.3 (10.8-14.0)	13.4 (11.6-15.4)
Late GI	67 (3.9)	4.5 (3.6-5.6)	4.5 (3.6-5.6)	4.5 (3.6-5.6)
Grade ≥3				
Acute GU	13 (0.6)	NA	NA	NA
Acute GI	2 (0.09)	NA	NA	NA
Late GU	46 (2.1)	1.8 (1.3-2.5)	2.4 (1.8-3.2)	3.2 (2.2-4.6)
Late GI	7 (0.3)	0.4 (0.2-0.8)	0.4 (0.2-0.8)	0.4 (0.2-0.8)

Abbreviations: GI, gastrointestinal; GU, genitourinary; NA, not applicable.

^a^ Toxic event scoring derived per institutional or clinical trial protocol, as described in the Methods.

^b^ One trial, from Georgetown University, had only grade 3 or greater toxic event data. Thus, the denominator for grade 2 toxic event incidence calculations is 1700 vs 2142 for grade 3.

Forty-two patients experienced late grade 3 GU toxic events, with 3 patients experiencing 2 separate late grade 3 GU toxic events and 1 patient subsequently developing a grade 4 GU toxic event, for an estimated 7-year cumulative incidence of grade 3 or higher GU toxic events of 2.4% (95% CI, 1.8%-3.2%) (Table 3). The median interval from SBRT to development of a grade 3 GU toxic event was 27 months (IQR, 18-61 months). Nine events (19.6%) occurred after 5 years of follow-up, and 2 events (4.3%) occurred after 6 years of follow-up. Of the 4 patients with grade 3 urinary frequency, 2 had undergone cystoscopy prior to SBRT and had experienced acute grade 3 urinary frequency. One patient with grade 3 hematuria had a papillary bladder tumor at 18 months. This tumor was ultimately thought to be the cause of the hematuria, but the time course was such that it was not deemed a secondary malignant neoplasm. The 1 grade 4 GU toxic event was an episode of hemorrhagic cystourethritis treated with multiple endoscopic treatments and bladder irrigation; this event occurred 1 month after dilation of a urethral stricture, which itself was detected 52 months after SBRT.

Six patients experienced late grade 3 GI toxic events and 1 patient developed a grade 4 GI toxic event, for an estimated 7-year cumulative incidence of grade 3 or higher GI toxic events of 0.4% (95% CI, 0.2%-0.8%) (Table 3). The median interval to development of a late grade 3 or higher toxic event was 31 months (IQR, 24-38 months). All events occurred within 5 years of SBRT. Of the 6 patients with grade 3 GI toxic events, 1 had a history of ulcerative colitis and a known dysplastic polyp outside the radiation field in the midsigmoid colon. This patient had hematochezia 24 months after SBRT, which was ultimately attributed to a colonic adenocarcinoma arising in the polyp. The patient with a grade 4 toxic event had an anal fistula 9 months after SBRT. This fistula arose in the context of prior diverticulitis, and an attempted surgical repair failed to correct the fistula. As the patient had occasional (monthly) perianal discharge, he deferred definitive repair with colostomy.

On multivariable logistic regression, only acute composite RTOG and CTCAE grade 3 or higher toxic events (GI or GU) were associated with late composite GI or GU RTOG and CTCAE grade 3 or higher toxic events (odds ratio, 19.42; 95% CI, 5.14-73.42; *P* = .008), while EQD_2_, fractionation, and treatment platform were not (eTables 7 and 8 in the Supplement). Both fractionation and acute composite RTOG and CTCAE grade 2 or higher toxic events (GI or GU) were associated with late composite GI or GU RTOG and CTCAE grade 2 or higher toxic events, with an odds ratio of 0.38 (95% CI, 0.16-0.89; *P* = .03) for fractionation and an odds ratio of 3.15 (95% CI, 1.96-5.07; *P* = .006) for acute composite RTOG and CTCAE grade 2 or higher toxic events.

## Discussion

In this individual patient data analysis of 2142 patients, SBRT was associated with a favorable long-term disease control and safety profile for low-risk and intermediate-risk PCa. Biochemical control was excellent, with 7-year BCR rates of less than 10% for low-risk and favorable intermediate-risk disease and just 15% for unfavorable intermediate-risk disease. Severe toxic events were rare in both the acute and late settings, with a 7-year cumulative incidence of late grade 3 or higher GU and GI toxic events of 2.4% and 0.4%. To our knowledge, these data represent the largest series of patients treated with SBRT for low-risk and intermediate-risk disease with long-term follow-up, and the cohort studied includes a substantial number of patients with follow-up extending beyond 9 years. Our findings suggest that the major trepidation with SBRT—a risk of severe late toxic events—is not supported even with mature follow-up data. Thus, the results of the present study directly address the statement within the NCCN guidelines that “longer follow-up and prospective multi-institutional data [following SBRT] are required to evaluate longer-term results, especially since late toxicity theoretically could be worse in hypofractionated regimens.”^2^^(pMS-17)^

**Figure 2.**
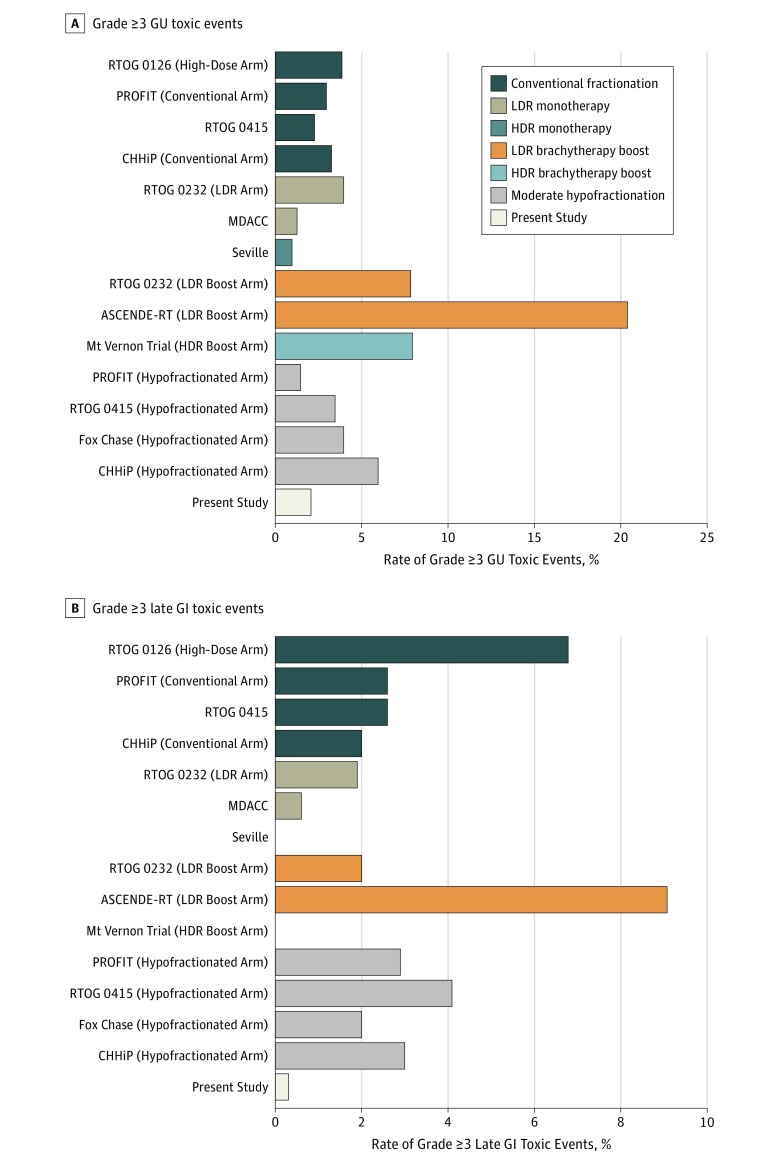
Comparative Rates of Grade 3 or Higher Toxic Events Across Various Radiotherapy Modalities A, Rate of grade 3 or higher genitourinary (GU) toxic events. B, Rate of grade 3 or higher late gastrointestinal (GI) toxic events. Additional details in eTable 9 in the Supplement. ASCENDE-RT indicates Androgen Suppression Combined With Elective Nodal and Dose Escalated Radiation Therapy; CHHiP, conventional vs hypofractionated high-dose intensity-modulated radiotherapy for prostate cancer; HDR, high-dose-rate brachytherapy; LDR, low dose-rate brachytherapy; MDACC, MD Anderson Cancer Center; PROFIT, Prostate Fractionated Irradiation Trial; and RTOG, Radiation Therapy Oncology Group.

Long-term outcomes are integral because patients with low-risk and intermediate-risk PCa have multiple other radiotherapy-based options for curative treatment, including conventionally fractionated EBRT, moderately hypofractionated EBRT, low-dose- or high-dose-rate brachytherapy as monotherapy, and conventionally fractionated EBRT with a brachytherapy boost (with the latter most often considered for unfavorable intermediate-risk disease only). To place the results of the present series in the appropriate context, we extracted the rates of late severe (ie, grade ≥3) toxic events after treatment with other radiotherapy modalities from prospective reports with long-term follow-up for each of the other modalities (Figure 2).^4,5,6,35,36,37,38,39,40,41^ Further information about these series can be found in eTable 9 in the Supplement. Overall, the outcomes after SBRT compare very favorably, without evidence of unanticipated late failures or increased late toxic effects.

This absence of significantly increased GU toxic events when compared with other radiotherapy modalities is in contradistinction to the findings of a Medicare claims–based analysis that included claims data from 1335 patients receiving SBRT (696 with at least 2 years of follow-up). Yu et al^13^ reported that significantly more patients treated with SBRT had claim codes indicative of treatment-related GU toxic events when compared with patients receiving intensity-modulated radiotherapy (IMRT), particularly with respect to diagnostic procedures for incontinence and obstruction. They also found significantly more claims indicative of diagnosis of or procedure to correct or investigate urethritis, urethral strictures, and obstruction among patients treated with SBRT. A potential limitation of such a population-based analysis, however, is that inferences are made upon amalgamating diagnostic and therapeutic intervention codes, leading to an overclassification of toxic events. A more recent analysis of claims in the MarketScan database found no significant differences in composite GU, GI, or erectile dysfunction toxic events between patients treated with SBRT or IMRT.^9^ The investigators did report a significant increase in claims pertaining to obstruction or retention (SBRT, 21% vs IMRT, 15%) and urinary fistula (SBRT, 1% vs IMRT, 0.1%) at 2 years. However, only 43 patients receiving SBRT were eligible for these end points at 2 years compared with 619 patients receiving IMRT; thus, these findings must be viewed with extreme caution given the very small numbers of patients included. On the other hand, the present study includes 2142 patients with individual patient data, and no patients had a urinary fistula and only 1 patient had a GI-related fistula (crude incidence, 0.05%). The most robust factors associated with late toxic events appear to be development of acute toxic events and fractionation, with treatment every other day associated with lower incidences of grade 2 or higher toxic events.

### Limitations

This study has some limitations. Although all patients were treated prospectively, the study still represents a consortium of multiple single-arm studies. Hence, in addition to selection bias related to enrollment—it is possible that the patients enrolled in these studies were at lower risk of a poor outcome or toxic event than the general patient with PCa—the study is limited by the lack of a direct comparator arm. Multiple ongoing randomized trials are directly comparing SBRT or extreme hypofractionation regimens with either conventional or moderate hypofractionated regimens and are thus better suited to conclude the true noninferiority of this approach. These trials include HYPO-RT-PC (ISRCTN45905321), PACE (NCT01584258), HEAT (NCT01794403), and NRG GU005 (NCT03367702). The preliminary results of the HYPO-RT-PC trial, which compared 78 Gy in 39 fractions with an extremely hypofractionated regimen of 42.7 Gy in 7 fractions, have been presented in abstract form.^42^ With a median follow-up of nearly 5 years, the proportion of patients free of biochemical or clinical failure at 5 years was 83.8% with conventional fractionation and 83.7% with extreme hypofractionation, suggesting noninferiority. There was no difference in modified late grade 2 or higher RTOG toxic events at 4 years with regard to either urinary or bowel axes. Although it is nonrandomized, the current consortium study provides prospective data supporting the safety of SBRT at even longer follow-up than in the HYPO-RT-PC study and also presents outcomes after more typical SBRT regimens than the 7-fraction course used in that trial. Given the low-risk and intermediate-risk nature of the disease that patients in this study had, it is likely that more clinical events (DMs or PCa-specific death) would be seen over time; however, as indicated in eTable 9 in the Supplement, the follow-up in the present study is among the longest for a series exploring radiotherapy modalities in this context.

Another limitation is that the present study includes only physician-scored toxic events, even though patient-reported outcomes may be more relevant, particularly for patients with low-risk and intermediate-risk PCa who have an excellent prognosis. Nonetheless, much of the aversion to SBRT is based on a preconceived risk of severe grade 3 or higher toxic events. These data suggest that this risk is minimal and commensurate with the risk after other, more widely accepted treatment modalities. Finally, the treatment protocols were heterogeneous, and there was no central review or quality assurance process.

## Conclusions

We present prospective, multi-institutional data with long-term follow-up demonstrating that SBRT for low-risk and intermediate-risk PCa is associated with a favorable safety and disease control profile. No unexpected increase in late toxic events or compromise in disease control was identified with longer-term follow-up (ie, beyond 5 years) than reported in prior studies. Although randomized trials comparing conventional or moderate hypofractionation regimens and SBRT are under way, the favorable outcomes described herein strongly suggest that SBRT be considered a standard option for treating low-risk and intermediate-risk PCa.

## Notes

Supplement. **eTable 1.** Specific Planning Considerations for Individual Studies **eTable 2.** Selected Overview of Toxicity Scales for Grade ≥3 Toxicity Events **eTable 3.** Cumulative Incidence or Kaplan-Meier Estimates for Selected End Points **eTable 4.** Competing Risk Regression Analysis for Predictors of Biochemical Recurrence **eTable 5.** Cox Proportional Hazards Regression Analysis for Predictors of Biochemical Recurrence **eTable 6.** Narrative Description of Severe Grade ≥3 Toxicity Outcomes **eTable 7.** Multivariable Logistic Regression for Predictors of Late Composite RTOG/CTCAE Grade ≥3 Toxicity **eTable 8.** Multivariable Logistic Regression for Predictors of Late Composite RTOG/CTCAE Grade ≥2 Toxicity **eTable 9.** Comparative Analysis of Efficacy and Safety of Radiation Treatment Options for Low- and Intermediate-Risk Prostate Cancer: An Analysis of Prospective Data With Long-term Follow-up **eReferences**
