# Supplementary material for: Long-term Outcomes of Stereotactic Body Radiotherapy for Low-Risk and Intermediate-Risk Prostate Cancer
Source: JAMA Netw Open. 2019 Feb 8;2(2):e188006. doi: 10.1001/jamanetworkopen.2018.8006 (PMC6484596; doi:10.1001/jamanetworkopen.2018.8006)
Supplement: Supplement. — eTable 1. Specific Planning Considerations for Individual Studies eTable 2. Selected Overview of Toxicity Scales for Grade ≥3 Toxicity Events eTable 3. Cumulative Incidence or Kaplan-Meier Estimates for Selected End Points eTable 4. Competing Risk Regression Analysis for Predictors of Biochemical Recurrence eTable 5. Cox Proportional Hazards Regression Analysis for Predictors of Biochemical Recurrence eTable 6. Narrative Description of Severe Grade ≥3 Toxicity Outcomes eTable 7. Multivariable Logistic Regression for Predictors of Late Composite RTOG/CTCAE Grade ≥3 Toxicity eTable 8. Multivariable Logistic Regression for Predictors of Late Composite RTOG/CTCAE Grade ≥2 Toxicity eTable 9. Comparative Analysis of Efficacy and Safety of Radiation Treatment Options for Low- and Intermediate-Risk Prostate Cancer: An Analysis of Prospective Data With Long-term Follow-up eReferences [file jamanetwopen-2-e188006-s001.pdf]

Supplementary Online Content

Kishan AU, Dang A, Katz AJ, et al. Long-term outcomes of stereotactic body radiotherapy for low-risk and intermediate-risk prostate cancer. *JAMA Netw Open.* 2019;2(2):e188006. doi:10.1001/jamanetworkopen.2018.8006

eTable 1. Specific Planning Considerations for Individual Studies

eTable 2. Selected Overview of Toxicity Scales for Grade ≥3 Toxicity Events

eTable 3. Cumulative Incidence or Kaplan-Meier Estimates for Selected End Points

eTable 4. Competing Risk Regression Analysis for Predictors of Biochemical Recurrence

eTable 5. Cox Proportional Hazards Regression Analysis for Predictors of Biochemical Recurrence

eTable 6. Narrative Description of Severe Grade ≥3 Toxicity Outcomes

eTable 7. Multivariable Logistic Regression for Predictors of Late Composite RTOG/CTCAE Grade ≥3 Toxicity

eTable 8. Multivariable Logistic Regression for Predictors of Late Composite RTOG/CTCAE Grade ≥2 Toxicity

eTable 9. Comparative Analysis of Efficacy and Safety of Radiation Treatment Options for Low- and Intermediate-Risk Prostate Cancer: An Analysis of Prospective Data With Long-term Follow-up

eReferences

This supplementary material has been provided by the authors to give readers additional information about their work.

© 2019 Kishan AU. *JAMA Network Open.*

eTable 1. Specific Planning Considerations for Individual Studies

Institution or Trial	Dose/Fraction	Prescription Specification	Margins	Planning Technique	Image Guidance
Virginia Mason	6·7 Gy x 5	90% of rx to cover 100% of prostate	4-5 mm from prostate to block edge	Six stationary noncoplanar fields	Orthogonal imaging to implanted fiducial markers prior to treatment
Stanford	7·25 Gy x 5	100% of rx to cover 95% of PTV	5 mm expansion from prostate, except 3 mm posteriorly	Noncoplanar fields (robotic gantry)	Real-time tracking of implanted fiducial markers
Flushing	7 Gy x 5 (32%)<br>7·25 Gy x 5 (68%)	100% of rx to cover 95% of PTV	5 mm expansion from prostate, except 3 mm posteriorly. SVs included for lesions at the base	Noncoplanar fields (robotic gantry)	Real-time tracking of implanted fiducial markers
21st Century Oncology	8 Gy x 5	100% of rx to cover 98% of PTV	2 mm isotropic expansion from prostate	7-9 non-opposing coplanar fields	Real-time tracking of implanted electromagnetic beacons
NCT00643994	7·25 Gy x 5	100% of rx to cover 95% of PTV	5 mm expansion from prostate, except 3 mm posteriorly. 2 cm of SVs included for int risk, same margins	Noncoplanar fields (robotic gantry)	Real-time tracking of implanted fiducial markers
NCT00643617	9·5 Gy x 4	100% of rx to cover 95% of PTV	2 mm expansion from prostate, except 0 mm posteriorly. 3 mm lateral extension for side(s) with Gleason 7 disease. 1 cm of SVs for int risk, same margins	Noncoplanar fields (robotic gantry)	Real-time tracking of implanted fiducial markers
Sunnybrook pHART 3	7 Gy x 5	95% of rx to cover 99% of PTV	4 mm isotropic expansion from prostate	"Step and shoot" intensity modulated radiotherapy	Orthogonal imaging to implanted fiducial markers prior to treatment
Sunnybrook pHART 6	8 Gy x 5	95% of rx to cover 99% of PTV	5 mm isotropic expansion from prostate	"Step and shoot" intensity modulated radiotherapy	Orthogonal imaging to implanted fiducial markers prior to treatment
Beth Israel Deaconess Medical Center	7·25 Gy x 5	100% of rx to cover 95% of PTV	5 mm expansion from prostate, except 3 mm posteriorly	Noncoplanar fields (robotic gantry)	Real-time tracking of implanted fiducial markers
University of California, Los Angeles	8 Gy x 5	100% of rx to cover 95% of PTV	5 mm expansion from prostate, except 3 mm posteriorly	Volumetric modulated arc therapy	Orthogonal imaging to implanted fiducial markers prior to and three times during treatment
Genesis Healthcare	9·5 Gy x 4	100% of rx to cover 95% of PTV	2 mm expansion from prostate, except 0 mm posteriorly. 3 mm lateral	Noncoplanar fields (robotic gantry)	Real-time tracking of implanted fiducial markers

© 2019 Kishan AU. *JAMA Network Open.*

			extension for side(s) with Gleason 7 disease. 1 cm of SVs for int risk, same margins		
Georgetown	7 Gy x 5 (33%)<br>7·25 Gy x 5 (67%)	100% of rx to cover 95% of PTV	5 mm expansion from prostate, except 3 mm posteriorly. Proximal SV up to bifurcation included.	Noncoplanar fields (robotic gantry)	Real-time tracking of implanted fiducial markers

© 2019 Kishan AU. *JAMA Network Open.*

eTable 2. Selected Overview of Toxicity Scales for Grade ≥3 Toxicity Events

	CTC 2.0	CTCAE v 3.0	CTCAE v4.0	RTOG (acute)	RTOG (late)
**Genitourinary**					
Urinary frequency/urgency		3: ≥1x/hour; urgency; catheter indicated	No grade 3	3: Frequency with urgency and nocturia hourly or more frequently	3: Severe frequency & dysuria
Urinary retention	3: requiring frequent in/out catheterization (≥4 x per week) or urological intervention (e.g., TURP, suprapubic tube, urethrotomy)<br>4: Bladder rupture	3: More than daily catheterization indicated; urological intervention indicated (e.g., TURP, suprapubic tube, urethrotomy)<br>4: Life-threatening consequences; organ failure (e.g., bladder rupture); operative intervention requiring organ resection indicated	3: Elective operative or radiologic intervention indicated; substantial loss of affected kidney function or mass<br>4: Life-threatening consequences; organ failure; urgent operative intervention indicated	4: acute bladder obstruction not secondary to clot passage, ulceration, or necrosis	
Hematuria	3: persistent gross bleeding or clots; may require catheterization or instrumentation, or transfusion<br>4: open surgery or necrosis or deep bladder ulceration	3: Transfusion, interventional radiology, endoscopic, or operative intervention indicated; radiation therapy (i.e., hemostasis of bleeding site)<br>4: Life-threatening consequences; major urgent intervention indicated	3: Gross hematuria; transfusion, IV medications or hospitalization indicated; elective endoscopic, radiologic or operative intervention indicated; limiting self care ADL<br>4: Life-threatening consequences; urgent radiologic or operative intervention indicated	3: gross hematuria with/without clot passage<br>4: Hematuria requiring transfusion	3: frequent hematuria<br>4: severe hemorrhagic cystitis
Stricture		3: Symptomatic and altered organ function (e.g., sepsis or hydronephrosis, or renal dysfunction);			

© 2019 Kishan AU. *JAMA Network Open.*

		operative intervention indicated<br>4: Life-threatening consequences; organ failure or operative intervention requiring organ resection indicated			
Incontinence	3: no control (in the absence of fistula)	3 Interfering with ADL; intervention indicated (e.g., clamp, collagen injections)<br>4: Operative intervention indicated (e.g., cystectomy or permanent urinary diversion)	3: Intervention indicated (e.g., clamp, collagen injections); operative intervention indicated; limiting self care ADL		3: reduction in bladder capacity (<150 cc)<br>4: Necrosis/contracted bladder (capacity < 100 cc)
Other				3: dysuria, pelvis pain or bladder spasm requiring regular, frequent narcotic	3: severe telangiectasia (often with petechiae)
**Gastrointestinal**					
Diarrhea	3: increase of ≥7 stools/day or incontinence; or need for parenteral support for dehydration<br>4: physiologic consequences requiring intensive care; or hemodynamic collapse	3: Increase of ≥7 stools per day over baseline; incontinence; IV fluids ≥24 hours; hospitalization; severe increase in ostomy output compared to baseline; interfering with ADL<br>4: Life-threatening consequences (e.g., hemodynamic collapse)	3: Increase of ≥7 stools per day over baseline; incontinence; hospitalization indicated; severe increase in ostomy output compared to baseline; limiting self care ADL<br>4: Life-threatening consequences; urgent intervention indicated	3: Diarrhea requiring parenteral support	
Bleeding	3: requiring transfusion<br>4: catastrophic bleeding, requiring major nonelective intervention	3: Interfering with ADL; interventional radiology, endoscopic, or operative intervention indicated	3: Transfusion, radiologic, endoscopic, or elective operative intervention indicated	3: Diarrhea requiring parenteral support<br>4: GI bleeding requiring transfusion	3: Obstruction or bleeding, requiring surgery

© 2019 Kishan AU. *JAMA Network Open.*

		4: Life-threatening consequences	4: Life-threatening consequences; urgent intervention indicated		
Fistula	3: present<br>4: requiring surgery	3: Symptomatic and severely altered GI function (e.g., altered dietary habits, diarrhea, or GI fluid loss); IV fluids, tube feedings, or TPN indicated ≥24 hrs<br>4: Life-threatening consequences	3: Severely altered GI function; TPN or hospitalization indicated; elective operative intervention indicated<br>4: Life-threatening consequences; urgent intervention indicated		4: Necrosis / perforation fistula

ADL, activity of daily living; CTC v2.0, common toxicity criteria version 2.0; CTCAE v3.0 or v4.0, common terminology criteria for adverse events; GI, gastrointestinal; RTOG, Radiation Therapy Oncology Group

© 2019 Kishan AU. *JAMA Network Open.*

eTable 3. Cumulative Incidence or Kaplan-Meier Estimates for Selected End Points

Risk Group	5-Year	7-Year	10-Year
Cumulative Incidence Estimate of Biochemical Recurrence (95% Confidence Interval)			
Low	2.5% (1.5%-3.4%)	4.5% (3.2%-5.8%)	8.6% (5.9%-11.2%)
Fav Int	6.3% (4.4%-8.3%)	8.6% (6.2%-11.0%)	13.5% (7.9%-19.1%)
Unfav Int	9.6% (5.7%-13.5%)	14.9% (9.5%-20.2%)	18.9% (12.1%-25.7%)
All Intermediate	7.2% (5.5%-9.0%)	10.2% (8.0%-12.5%)	15.0% (10.3%-19.7%)
Cumulative Incidence Estimate of Distant Metastasis (95% Confidence Interval)			
Low	0.1% (0.0%-0.3%)	0.1% (0.0%-0.3%)	0.5% (0.0%-1.2%)
Fav Int	1.5% (0.5%-2.4%)	1.7% (0.6%-2.8%)	1.7% (0.6%-2.8%)
Unfav Int	1.8% (0.0%-3.6%)	3.0% (0.1%-5.8%)	3.0% (0.1%-5.8%)
All Intermediate	1.6% (0.7%-2.4%)	2.0% (1.0%-3.0%)	2.0% (1.0%-3.0%)
Kaplan-Meier Estimate of Biochemical Recurrence-Free Survival (95% Confidence Interval)			
Low	92.2% (90.4%-93.6%)	87.2% (85.0%-89.2%)	79.6% (75.6%-83.0%)
Fav Int	90.5% (87.8%-92.6%)	85.4% (81.9%-88.2%)	78.0% (71.2%-83.4%)
Unfav Int	82.8% (77.3%-87.2%)	74.3% (67.2%-80.1%)	70.3% (62.0%-77.1%)
All Intermediate	88.4% (86.0%-90.4%)	82.4% (79.3%-85.1%)	75.8% (70.2%-80.4%)
Kaplan-Meier Estimate of Overall Survival (95% Confidence Interval)			
Low	94.5% (93.0%-95.7%)	91.4% (89.4%-93.0%)	87.6% (84.5%-90.1%)
Fav Int	96.7% (94.8%-97.9%)	93.7% (91.0%-95.6%)	91.0% (87.4%-93.6%)
Unfav Int	92.0% (87.6%-94.8%)	86.5% (80.6%-90.7%)	86.5% (80.6%-90.7%)
All Intermediate	95.4% (93.7%-96.6%)	91.7% (89.2%-93.6%)	89.6% (86.6%-92.0%)

Fav Int, favorable intermediate; Unfav Int, unfavorable intermediate

© 2019 Kishan AU. *JAMA Network Open.*

eTable 4. Competing Risk Regression Analysis for Predictors of Biochemical Recurrence

	**Low Risk**		**Favorable Intermediate Risk**		**Unfavorable Intermediate-Risk**	
	SHR (95% CI)	p-value	SHR (95% CI)	p-value	SHR (95% CI)	p-value
**Age**	1.00 (0.97-1.03)	0.80	1.01 (0.99-1.03)	0.47	1.00 (0.98-1.03)	0.77
**T stage**						
**1c**	Reference		Reference		Reference	
**2a**	0.79 (0.46-1.35)	0.39	1.23 (0.62-2.45)	0.55	**3.15 (1.22-8.13)**	**0.02**
**2b**			1.45 (0.70-3.03)	0.32	**4.92 (2.19-11.22)**	**<0.01**
**2c**			**2.88 (1.31-6.34)**	**<0.01**	2.54 (0.76-8.48)	0.13
**ln(initial PSA)**	1.11 (0.42-2.95)	0.83	1.98 (0.75-5.23)	0.17	1.10 (0.59-2.05)	0.77
**Gleason Grade group**						
**I**	Reference		Reference		Reference	
**II**			0.78 (0.38-1.61)	0.5	**46011.52 (7874.75-268841.64)**	**<0.01.**
**III**					**47043.18 (9950.10-222415.82)**	**<0.01**
**EQD_2 (≤91 Gy vs. >91 Gy)***	0.99 (0.96-1.01)	0.30	1.03 (0.99-1.08)	0.18	0.94 (0.86-1.04)	0.23
**ADT Use**	1.03 (0.37-2.85)	0.95	0.46 (0.17-1.27)	0.13	0.88 (0.70-1.10)	0.26

*Refers to the "equivalent dose in 2 Gy fractions", and is calculated assuming an α/Æ ratio of 1.5. 7.25 Gy in 5 fractions delivers an EQD_2 of ~91 Gy.
95% CI, 95% confidence interval; ADT, androgen deprivation therapy; EQD_2, equivalent dose in 2 Gy fractions; SHR, subdistribution hazard ratio

© 2019 Kishan AU. *JAMA Network Open.*

eTable 5. Cox Proportional Hazards Regression Analysis for Predictors of Biochemical Recurrence

	**Low Risk**		**Favorable Intermediate Risk**		**Unfavorable Intermediate-Risk**	
	HR (95% CI)	p-value	HR (95% CI)	p-value	HR (95% CI)	p-value
**Age**	1.00 (0.96-1.03)	0.94	1.01 (0.97-1.05)	0.57	1.01 (0.97-1.06)	0.62
**T stage**						
**1c**	Reference		Reference		Reference	
**2a**	0.79 (0.35-1.78)	0.57	1.25 (0.55-2.85)	0.59	**3.40 (1.40-8.27)**	**<0.01**
**2b**			1.50 (0.52-4.35)	0.46	**4.48 (1.22-16.40)**	**0.02**
**2c**			2.75 (0.27-27.99)	0.39	4.39 (0.43-44.40)	0.21
**ln(initial PSA)**	1.15 (0.65-2.05)	0.63	1.96 (0.93-4.14)	0.08	1.16 (0.56-2.40)	0.69
**Gleason Grade group**						
**I**	Reference		Reference		Reference	
**II**			0.77 (0.37-1.60)	0.48	>100000 (0-Inf)	1.00
**III**					>100000 (0-Inf)	1.00
**EQD_2 (≤91 Gy vs. >91 Gy)***	0.99 (0.92-1.06)	0.74	1.03 (0.96-1.11)	0.38	0.94 (0.86-1.02)	0.14
**ADT Use**	1.02 (0.31-3.41)	0.97	0.48 (0.11-2.06)	0.32	0.96 (0.31-2.97)	0.94

*Refers to the "equivalent dose in 2 Gy fractions", and is calculated assuming an α/Æ ratio of 1.5. 7.25 Gy in 5 fractions delivers an EQD_2 of ~91 Gy.

95% CI, 95% confidence interval; ADT, androgen deprivation therapy; EQD_2, equivalent dose in 2 Gy fractions; HR, hazard ratio

© 2019 Kishan AU. *JAMA Network Open.*

eTable 6. Narrative Description of Severe Grade ≥3 Toxicity Outcomes

**ACUTE URINARY TOXICITIES**
Frequency (8)
Grade 3 frequency at one month, which resolved but recurred at 21 months.
Grade 3 frequency at one month.
Grade 3 frequency at one month.
Grade 3 frequency at one month.
Grade 3 frequency at one month. Had cystoscopic evaluation prior to SBRT
Grade 3 frequency at one month.
Grade 3 frequency at one month, which resolved but recurred at six months.
Grade 3 frequency at one month, which resolved but recurred at six months. Had cystoscopic evaluation prior to SBRT.
Retention (4)
Grade 3 retention after first fraction requiring temporary indwelling catheter.
Grade 3 retention after second fraction requiring temporary indwelling catheter.
Grade 3 retention at two weeks requiring temporary indwelling catheter.
Grade 3 retention at one month requiring temporary indwelling catheter.
Hematuria (1)
Gross hematuria 1 month after SBRT. Cystoscopy at two months showed urethritis.
**LATE URINARY TOXICITIES**
Frequency (4)
Grade 3 frequency and dysuria at 6 months. Had cystoscopic evaluation prior to SBRT
Grade 3 frequency at 6 months. Had cystoscopic evaluation prior to SBRT.
Grade 3 frequency at 6 months.
Grade 3 frequency at 21 months.
Retention (17)
Grade 3 retention at 12 months, treated with TURP
Grade 3 retention at 12 months, treated with TURP
Grade 3 retention at 14 months, treated with TURP
Grade 3 retention at 16 months, treated with TURP
Grade 3 retention at 17 months, treated with TURP
Grade 3 retention at 18 months, treated with TURP
Grade 3 retention at 18 months, treated with TURP
Grade 3 retention at 20 months, treated with TURP
Grade 3 retention at 32 months, treated with TURP

© 2019 Kishan AU. *JAMA Network Open.*

Grade 3 retention at 38 months, treated at 42 months with direct visual internal urethrotomy for bladder neck contracture.
Grade 3 retention at 44 months, treated with TURP
Grade 3 retention at 50 months, treated with TURP
Grade 3 retention at 68 months, treated with TURP
Grade 3 retention at 69 months, treated with TURP
Grade 3 retention at 77 months, treated with TURP
Grade 3 retention at 84 months, treated with TURP
Grade 3 retention at 89 months, treated with TURP
Hematuria (12)
Grade 3 hematuria at 6 months.
Grade 3 hematuria at 12 months. Found to have stricture on cystoscopy.[x1]
Grade 3 hematuria at 18 months.
Grade 3 hematuria, cauterized at 18 months.
Grade 3 hematuria at 18 months. Found on cystoscopy to have a papillary bladder tumor.
Grade 3 hematuria at 24 months, treated with laser coagulation.
Grade 3 hematuria at 24 months, treated with laser coagulation.
Grade 3 hematuria at 25 months.
Grade 3 hematuria at 49 months later, also needed dilation of stricture at 72 months.[x2]
Grade 3 hematuria at 72 months.
Grade 3 hematuria at 75 months.
Grade 3 hematuria at 108 months.
Stricture (7)
Grade 3 hematuria at 12 months. Found to have stricture on cystoscopy. [x1]
Developed urethral stricture at 24 months.
Developed urethral stricture at 24 months. Subsequently underwent biopsy for suspected local recurrence at 36 months and developed incontinence. Biopsy did confirm local recurrence.[x3]
Developed stricture at 42 months.
Developed urethral stricture at 52 months, which was dilated. Subsequently developed grade 4 hemorrhagic cystourethritis 1 month after dilation.[x4]
Dilation of stricture at 72 months, also had gross hematuria at 49 months.[x2]

© 2019 Kishan AU. *JAMA Network Open.*

Found to have urethral stricture in membranous urethra on cystoscopy done at 76 months; also had hypotonic bladder from poorly controlled diabetes mellitus.
Incontinence (4)
Urge incontinence at 12 months, needed artificial sphincter.
Developed urethral stricture at 24 months. Subsequently underwent biopsy for suspected local recurrence at 36 months and developed incontinence. Biopsy did confirm local recurrence.[x3]
Overflow incontinence at 27 months, ultimately leading to urethral dilation and ultimately a dorsal slit with bladder neck incision at 39 months.
Urge incontinence at 84 months, needed artificial sphincter.
Other Severe Toxicities (2)
Bladder neck necrosis
Hemorrhagic cystourethritis one month after dilation of stricture, which took place 52 months after SBRT. [x4]
Toxicities After Salvage Procedures (3)
Developed local recurrence at 36 months and underwent salvage RP at 38 months. Immediately after this, had significant incontinence and developed a bladder neck contraction requiring bladder neck incision and artificial sphincter placement.
Developed local recurrence at 52 months and underwent salvage HDR at 60 months. Developed prostatic abscess and subsequent urinary incontinence.
Developed local recurrence at 54 months and had salvage LDR at 58 months. Stricture developed 50 months later (i.e., 108 months after SBRT)
**ACUTE GI TOXICITIES**
Diarrhea (2)
Grade 3 diarrhea at week 2
Grade 3 diarrhea at week 2
**LATE GI TOXICITIES**
Bleeding (5)
Grade 3 hematochezia at 6 months, treated with argon plasma coagulation.
Grade 3 hematochezia at 6 months, treated with argon plasma coagulation.
Grade 3 hematochezia at 30 months, treated with argon plasma coagulation. Continued to have bleeding, and ultimately underwent hemicolectomy, which identified a colonic adenocarcinoma.
Grade 3 hematochezia at 34 months, treated with formalin.
Grade 3 hematochezia at 49 months, treated with argon plasma coagulation.
Fistula (1)
Developed a fistula in ano at 9 months. Had a history of diverticulitis, for which he had refused management on prior occasions.
Other Severe Toxicities (1)

© 2019 Kishan AU. *JAMA Network Open.*

Had history of ulcerative colitis and known dysplastic polyp near the sigmoid colon prior to SBRT. Had hematochezia at 24 months, and was found to have a colon cancer arising in this polyp, requiring a proctocolectomy.
Toxicities After Salvage Procedures (1)
Developed local recurrence 65 months after SBRT and underwent salvage HDR at 69 months. Developed prostatic abscess and then a rectoprostatic fistula 6 months later (75 months after SBRT).

[1-4]Footnotes intended to track patients who had multiple toxicity events (i.e., both entries followed by footnote "1" refer to the same

© 2019 Kishan AU. *JAMA Network Open.*

eTable 7. Multivariable Logistic Regression for Predictors of Late Composite RTOG/CTCAE Grade ≥3 Toxicity

Parameter	Odds Ratio (95% CI)	p-value
EQD_2 (≤91 Gy vs. >91 Gy)*	0.99 (0.98-1.01)	0.41
Fractionation (every other day versus daily)**	0.54 (0.21-1.38)	0.20
Treatment Platform (gantry-mounted linear accelerator versus CyberKnife)	1.06 (0.33-3.38)	0.93
Acute Composite RTOG/CTCAE Grade ≥3 toxicity	**19.42 (5.14-73.42)**	**<0.01**

*Refers to the "equivalent dose in 2 Gy fractions", and is calculated assuming an α/Æ ratio of 1.5. 7.25 Gy in 5 fractions delivers an EQD_2 of ~91 Gy.

**Weekly fractionation was not included because the data came from a single center

95% CI, 95% confidence interval

© 2019 Kishan AU. *JAMA Network Open.*

eTable 8. Multivariable Logistic Regression for Predictors of Late Composite RTOG/CTCAE Grade ≥2 Toxicity

**Parameter**	**Odds Ratio (95% CI)**	**p-value**
EQD_2 (≤91 Gy vs. >91 Gy)*	1.00 (0.98-1.03)	0.76
Fractionation (every other day versus daily)**	**0.38 (0.16-0.89)**	**0.03**
Treatment Platform (linear accelerator versus CyberKnife)	1.39 (0.25-7.93)	0.71
Acute Composite RTOG/CTCAE Grade ≥2	**3.15 (1.96-5.07)**	**<0.01**

*Refers to the "equivalent dose in 2 Gy fractions", and is calculated assuming an α/Æ ratio of 1.5. 7.25 Gy in 5 fractions delivers an EQD_2 of ~91 Gy.

**Weekly fractionation was not included because the data came from a single center

95% CI, 95% confidence interval

© 2019 Kishan AU. *JAMA Network Open.*

eTable 9. Comparative Analysis of Efficacy and Safety of Radiation Treatment Options for Low- and Intermediate-Risk Prostate Cancer: An Analysis of Prospective Data With Long-term Follow-up

**Study**	**Number of Patients**	**Dose**	**Risk Groups**	**Median Follow-up (Years)**	**Biochemical Control Endpoint**	**Acute GU Toxicity**	**Acute GI Toxicity**	**Late GU Toxicity**	**Late GI Toxicity**
**SBRT**									
Present Study	2142	33.5-40 Gy in 4-5 fractions	56.0% Low<br>31.5% Fav Int<br>12.5% Unfav Int	6.9	5-year BCR<br>2.5% Low<br>6.3% Fav Int<br>9.6% Unfav Int<br>7.2% Int<br><br>7-year BCR<br>4.5% Low<br>8.6% Fav Int<br>14.9% Unfav Int<br>10.2% Int	RTOG/CTCAE<br>3: 0.6%<br>4: 0%	RTOG/CTCAE<br>3: 0.09%<br>4: 0%	RTOG/CTCAE<br>3: 2.1%%<br>4: 0.05%	RTOG/CTCAE<br>3: 0.4%<br>4: 0%
**Conventional Fractionation**									
RTOG 0126, high dose arm[1]	728-736*	79.2 Gy (34.4% IMRT)	100% Int	8.4	5-year BCR: 13%<br>8-year BCR: 20%	CTC v2.0<br>3: 1%	CTC v2.0<br>3: <1%	RTOG/EORTC<br>3: 3%<br>4: <1%	RTOG/EORTC<br>3: 5%<br>4: <1%<br>5: <1%
PROFIT, conventional arm[2]	598	78 Gy (most with IMRT)	100% Int	6.0	5-year BCRFS 85%	RTOG<br>3: 4.0%<br>4: 0%	RTOG<br>3 0.5%<br>4: 0%	RTOG<br>3: 2.8%<br>4: 0.2%	RTOG<br>3: 2.7%<br>4: 0.2%
RTOG 0415, conventional arm[3]	542	73.8 Gy (IMRT in 78.7%)	100% Low	5.8	5-year BCR: 8.1%	RTOG<br>3: 2.4%<br>4: 0%	RTOG<br>3: 0.6%<br>4: 0%	RTOG<br>3: 2.1%<br>4: 0.2%	RTOG<br>3: 2.4%<br>4: 0.2%
CHHiP, conventional arm[4]	1065	74 Gy	15% Low<br>73% Int<br>12% High	5.2	5-year BCFFS: 88.3%			RTOG<br>3: 3%	RTOG<br>3: 2%
**LDR Brachytherapy as Monotherapy**									
RTOG 0232, LDR arm[5]	292	125-145 Gy	100% Int	6.7	5-year FFP: 85.6%	CTC v2.0<br>3: 5%<br>4: 0%	CTC v 2.0<br>3: 1%<br>4: 0%	RTOG/EORTC<br>3: 3%<br>4: 1%	RTOG/EORTC<br>3: 1%<br>4: <1%
MD Anderson Cancer Center[6]	300	115-145 Gy	100% Int	5.1	5-year BCRFS: 92.7%			CTCAE v 4.0<br>3: 1.3%	CTCAE v 4.0<br>3: 0.6%
**HDR Brachytherapy Monotherapy**									
Seville[7]	119	13.5 Gy in 2 fractions	71% Low<br>29% Int	4.4	Actuarial FFBCR 98%	CTCAE v3.0<br>3: 2%	CTCAE v3.0<br>3: 0%	CTCAE v3.0<br>3:1%	CTCAE v3.0<br>3: 0
**Conventional Fractionation with LDR Brachytherapy Boost**									
RTOG 0232, LDR boost arm[5]	287	45 Gy + 100-110 Gy (43% IMRT)	100% Int	6.7	5-year FFP: 84.5%	CTCAE v2.0<br>3: 5%<br>4: 0%	CTCAE v 2.0<br>3: <1%<br>4: <1%	RTOG/EORTC<br>3: 7%<br>4: <1%	RTOG/EORTC<br>3: 2%<br>4: 0%
ASCENDE-RT, LDR boost arm[8,9]	198	46 Gy + 115 (0% IMRT)	29.8% Int<br>70.2% High	6.5	5-year BCRFS: 88.7%<br>7-year BCRFS: 86.2%	LENT/SOMA<br>3: 2.5%	LENT/SOMA<br>3: 0%	LENT/SOMA<br>3: 18.4%<br>4-5: 2.1%	LENT/SOMA<br>3: 8.1%<br>4-5: 1.0%

© 2019 Kishan AU. *JAMA Network Open.*

**Conventional Fractionation with HDR Brachytherapy Boost**									
Mt Vernon Trial, HDR boost arm[10]	110	35.75 Gy in 13 fractions + 8.5 Gy x 2 (0% IMRT)	2% Low<br>44% Int<br>54% High	7.1	7-year FFBCR<br>66% Int<br>46% High			Dische<br>7-year urethral stricture: 8%	
**Moderate Hypofractionation**									
PROFIT, hypofractionated arm[2]	608	60 Gy in 20 fractions	100% Int	6.0	5-year BCRFS: 85%	RTOG<br>3: 3.9%<br>4: 0%	RTOG<br>3: 0.7%<br>4: 0%	RTOG<br>3: 1.5%<br>4: 0%	RTOG<br>3: 2.7%<br>4: 0.2%
RTOG 0415, hypofractionated arm[3]	550	70 Gy in 28 fractions (IMRT in 79.6%)	100% Low	5.8	5-year BCR: 6.3%	RTOG<br>3: 3.3%<br>4: 0%	RTOG<br>3: 0.6%<br>4: 0.2%	RTOG<br>3: 3.5%<br>4: 0.0%	RTOG<br>3: 4.1%<br>4: 0.0%
ChHIP, hypofractionated arm[4]	1074	60 Gy in 20 fractions	15% Low<br>73% Int<br>12% High	5.2	5-year BCFFS: 90.6%			RTOG<br>3: 6%	RTOG<br>3: 3%

BCFFS, biochemical-clinical failure-free survival; BCR, biochemical recurrence; BCRFS, biochemical recurrence-free survival; CET, California Endocurie Therapy; CTC v2.0, common toxicity criteria version 2.0; CTCAE v3.0 or v4.0, common terminology criteria for adverse events; EORTC, European Organization for Research and Treatment of Cancer; FFBCR, freedom from biochemical recurrence; FFP, freedom from progression; HDR, high dose rate brachytherapy; IMRT, intensity modulated radiotherapy; LDR, low dose rate brachytherapy; LENT, late effects of normal tissue; RTOG, radiation therapy oncology group; TURP, transurethral resection of the prostate; SBRT, stereotactic body radiotherapy

© 2019 Kishan AU. *JAMA Network Open.*

eReferences

1. Michalski JM, Moughan J, Purdy J, et al. Effect of Standard vs Dose-Escalated Radiation Therapy for Patients With Intermediate-Risk Prostate Cancer: The NRG Oncology RTOG 0126 Randomized Clinical Trial. *JAMA oncology* 2018.

2. Catton CN, Lukka H, Gu CS, et al. Randomized Trial of a Hypofractionated Radiation Regimen for the Treatment of Localized Prostate Cancer. *Journal of clinical oncology : official journal of the American Society of Clinical Oncology* 2017; **35**(17): 1884-90.

3. Lee WR, Dignam JJ, Amin MB, et al. Randomized Phase III Noninferiority Study Comparing Two Radiotherapy Fractionation Schedules in Patients With Low-Risk Prostate Cancer. *Journal of clinical oncology : official journal of the American Society of Clinical Oncology* 2016; **34**(20): 2325-32.

4. Dearnaley D, Syndikus I, Mossop H, et al. Conventional versus hypofractionated high-dose intensity-modulated radiotherapy for prostate cancer: 5-year outcomes of the randomised, non-inferiority, phase 3 CHHiP trial. *The Lancet Oncology* 2016; **17**(8): 1047-60.

5. Prestidge BR, Winter K, Sanda MG, et al. Initial Report of NRG Oncology/RTOG 0232: A Phase 3 Study Comparing Combined External Beam Radiation and Transperineal Interstitial Permanent Brachytherapy With Brachytherapy Alone for Selected Patients With Intermediate-Risk Prostatic Carcinoma. *International Journal of Radiation Oncology • Biology • Physics* 2016; **96**(2): S4.

6. Frank SJ, Pugh TJ, Blanchard P, et al. Prospective Phase 2 Trial of Permanent Seed Implantation Prostate Brachytherapy for Intermediate-Risk Localized Prostate Cancer: Efficacy, Toxicity, and Quality of Life Outcomes. *International journal of radiation oncology, biology, physics* 2018; **100**(2): 374-82.

7. Nagore G, Lopez Guerra JL, Krumina E, et al. High dose rate brachytherapy for prostate cancer: A prospective toxicity evaluation of a one day schedule including two 13.5Gy fractions. *Radiotherapy and oncology : journal of the European Society for Therapeutic Radiology and Oncology* 2018; **127**(2): 219-24.

8. Morris WJ, Tyldesley S, Rodda S, et al. Androgen Suppression Combined with Elective Nodal and Dose Escalated Radiation Therapy (the ASCENDE-RT Trial): An Analysis of Survival Endpoints for a Randomized Trial Comparing a Low-Dose-Rate Brachytherapy Boost to a Dose-Escalated External Beam Boost for High- and Intermediate-risk Prostate Cancer. *International journal of radiation oncology, biology, physics* 2017; **98**(2): 275-85.

9. Rodda S, Tyldesley S, Morris WJ, et al. ASCENDE-RT: An Analysis of Treatment-Related Morbidity for a Randomized Trial Comparing a Low-Dose-Rate Brachytherapy Boost with a Dose-Escalated External Beam Boost for High- and Intermediate-Risk Prostate Cancer. *International journal of radiation oncology, biology, physics* 2017; **98**(2): 286-95.

10. Hoskin PJ, Rojas AM, Bownes PJ, Lowe GJ, Ostler PJ, Bryant L. Randomised trial of external beam radiotherapy alone or combined with high-dose-rate brachytherapy boost for localised prostate cancer. *Radiotherapy and oncology : journal of the European Society for Therapeutic Radiology and Oncology* 2012; **103**(2): 217-22.

© 2019 Kishan AU. *JAMA Network Open.*
